# Reinforced Capacity and Cycling Stability of CoTe Nanoparticles Anchored on Ti_3_C_2_ MXene for Anode Material

**DOI:** 10.1002/smtd.202500725

**Published:** 2025-07-01

**Authors:** Ramesh Subramani, Su‐Yang Hsu, Wei‐Hsiang Huang, Zhiwei Hu, Kueih‐Tzu Lu, Jin‐Ming Chen

**Affiliations:** ^1^ Department of Electrophysics National Yang Ming Chiao Tung University (NYCU) Hsinchu 300093 Taiwan; ^2^ National Synchrotron Radiation Research Center (NSRRC) Hsinchu 300092 Taiwan; ^3^ Max Planck Institute for Chemical Physics of Solids 01187 Dresden Germany

**Keywords:** CoTe, in situ XAS, in situ XRD, LIBs, MXene, Ti_3_C_2_

## Abstract

Developing high‐performance anode materials is critical for lithium‐ion batteries (LIBs) to meet consumers' demands. Cobalt tellurides (CoTe) exhibit promising electrochemical properties due to their higher theoretical capacity compared to commonly used graphite anodes. However, their practical application is hindered by poor electrical conductivity, agglomeration of nanoparticles, and significant volume changes during charge‐discharge cycling. To overcome these challenges, CoTe nanoparticles are synthesized and anchored on Ti_3_C_2_ MXene (CoTe@Ti_3_C_2_) via a facile hydrothermal approach. The integration of CoTe nanoparticles with Ti_3_C_2_ nanosheets leverages their synergistic advantages: Ti_3_C_2_ MXene serves as a conductive substrate, improving electrical conductivity, reducing CoTe agglomeration, and accommodating volume changes, while CoTe nanoparticles prevent Ti_3_C_2_ nanosheet restacking. As a result, compared to the CoTe electrode, the CoTe@Ti_3_C_2_ anode exhibits an exceptional capacity increase, exceeding tenfold and reaching 698 mAh g^−1^ after 1000 cycles at 0.1 A g^−1^. Additionally, the CoTe@Ti_3_C_2_ anode demonstrates long‐term cycling stability over 1300 cycles at 1 A g^−1^. In situ synchrotron X‐ray diffraction and in situ X‐ray absorption spectroscopy elucidate the insights into the charge storage mechanisms. The superior electrochemical performance of CoTe@Ti_3_C_2_ highlights its potential as a high‐performance anode material for next‐generation LIBs.

## Graphical abstract

A Cobalt telluride (CoTe) nanoparticle‐anchored Ti_3_C_2_ MXene composite (CoTe@Ti_3_C_2_), synthesized via a hydrothermal approach, enhances electrochemical performance by leveraging the synergy between CoTe nanoparticles and Ti_3_C_2_ MXene nanosheets. Ti_3_C_2_ MXene enhances conductivity and structural stability, while CoTe nanoparticles prevent nanosheet restacking. This results in extended cycling stability and high capacity, making it a promising anode material for lithium‐ion batteries.

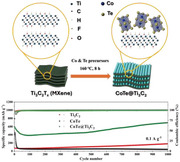

## 1 Introduction

With the increasing dependency on fossil fuels and the ever‐growing global demand for energy, the need for clean and environmentally sustainable renewable energy storage alternatives has become imperative.^[^ ^1^, ^2^, ^3^ ^]^ Rechargeable lithium‐ion batteries (LIBs) are currently the dominant power source for portable electronic devices and electric vehicles due to their high energy density and environmental benefits.^[^ ^4^, ^5^ ^]^ However, traditional LIBs with graphite anodes face limitations in terms of capacity and energy density, making it challenging to meet rising consumer demand.^[^ ^6^, ^7^ ^]^ To overcome these limitations, alternative battery technologies such as sodium‐ion batteries (SIBs) and potassium‐ion batteries have gained attention due to the abundance and low cost.^[^ ^8^, ^9^, ^10^, ^11^ ^]^ However, the larger ionic radii of Na^+^ and K^+^ lead to sluggish ion diffusion, greater volume changes, and reduced energy density, thereby limiting rate performance and cycle life.^[^ ^12^ ^]^ As a result, extensive research efforts has been dedicated to developing versatile anode materials for next‐generation LIBs.^[^ ^13^ ^]^ A variety of anode materials, including metal oxides,^[^ ^7^, ^14^, ^15^ ^]^ alloy anodes,^[^ ^16^, ^17^ ^]^ and transition metal chalcogenides (TMCs).^[^ ^18^, ^19^, ^20^ ^]^ have been explored. These anode materials operate via different charge storage mechanisms such as insertion, alloying, conversion, and conversion‐alloying processes. Among them, conversion‐type anodes show promise due to their high theoretical capacity and relatively simple redox reactions.^[^ ^21^, ^22^, ^23^, ^24^ ^]^ TMCs have gained considerable attention due to their unique properties and higher theoretical capacities compared to graphite.^[^ ^19^, ^25^ ^]^ Among the chalcogens, tellurium (Te) stands out for its remarkable characteristics, including high intrinsic conductivity (≈2 × 10^2^ S cm^−1^), and lower electronegativity (2.0), resulting in excellent rate capability and improved cycling stability. Additionally, among TMCs, metal tellurides have drawn substantial attention due to their relatively weaker M─Te bonds, which enhance electrode kinetics.^[^ ^26^, ^27^ ^]^

Cobalt telluride compounds (Co_x_Te_y_) have been widely studied for many applications, including catalysts for hydrogen/oxygen evolution reactions and energy storage devices.^[^ ^28^, ^29^, ^30^, ^31^, ^32^, ^33^, ^34^ ^]^ This is largely due to their diverse stoichiometric compositions (CoTe, CoTe_2,_ and Co_2_Te_3_) and unique crystal structures (hexagonal, orthorhombic, and hexagonal/rhombohedral).^[^ ^35^ ^]^ Despite these advantages, their use in energy storage applications is hindered by poor electrical conductivity, agglomeration of nanoparticles, and significant volume changes during charge‐discharge cycles, which lead to a rapid decline in capacity and significant structural collapse of the electrode materials.^[^ ^36^, ^37^, ^38^ ^]^ To overcome these challenges, researchers have explored modification strategies such as developing composite structures and introducing defects.^[^ ^28^, ^36^, ^39^, ^40^ ^]^ For example, Zhang et al. (2018) synthesized CoTe_2_/graphene composites via a one‐pot solvothermal method for Na^+^ storage, achieving enhanced cycling stability.^[^ ^41^ ^]^ Similarly, Ganesan et al. (2020) reported a polyhedral CoTe_2_‐C nanocomposite as an anode material for both LIBs and SIBs, demonstrating stable cycling performance up to 200 cycles.^[^ ^42^ ^]^ Unfortunately, the challenges associated with Co_x_Te_y_ remain major obstacles for their practical application in LIBs.

MXenes, a novel class of 2D transition metal carbides and nitrides, with the chemical formula M_n+1_X_n_T_x_, where M represents a transition metal (Sc, Ti, V, Cr, Zr, Nb, Mo, Hf, Ta), X is either carbon or nitrogen, n is an integer ranging from 1 to 3, and T_x_ denotes surface terminations (−OH, −F, and = O).^[^ ^43^, ^44^, ^45^, ^46^ ^]^ MXenes are synthesized by selectively etching the “A” layer from the MAX phases, resulting in layered 2D materials structurally similar to graphene, inspiring the name “MXene.” Since the discovery of Ti_3_C_2_ in 2011, more than 30 types of MXenes have been developed from a wide variety of MAX phase compounds.^[^ ^47^ ^]^ The versatile chemistry of MXenes allows fine‐tuning of their properties for various applications, including catalysts,^[^ ^48^, ^49^ ^]^ energy storage,^[^ ^50^, ^51^ ^]^ sensing,^[^ ^52^, ^53^ ^]^ water purification,^[^ ^54^, ^55^ ^]^ and photocatalysts for hydrogen production.^[^ ^56^ ^]^ Due to their large interlayer spacing, excellent conductivity (≈2 × 10^4^ S cm^−1^), low energy barrier for ion diffusion, and good mechanical properties, MXenes are well‐suited for energy storage applications.^[^ ^57^, ^58^ ^]^ However, their electrochemical performance and storage capacity are significantly lower than theoretical values. This is attributed to the high surface energy of MXene nanosheets, which reduces the interlayer gap due to Van der Waals interactions, leading to nanosheet restacking, as well as the presence of surface‐terminated functional groups.^[^ ^59^, ^60^ ^]^ To this end, the construction of composite materials with MXene has been shown to enhance electrochemical performance and structural stability.^[^ ^60^, ^61^, ^62^ ^]^

In this study, we developed a composite structure by integrating CoTe nanoparticles and Ti_3_C_2_ MXene nanosheets (denoted as CoTe@Ti_3_C_2_) for Li storage applications using a simple hydrothermal route. To the best of our knowledge, the CoTe@Ti_3_C_2_ composite has not been reported before for LIBs. The synergistic effect between CoTe and Ti_3_C_2_ MXene enhances the overall structural stability and significantly improves electrochemical performance. Ti_3_C_2_ MXene, acts as a conductive substrate, improving overall electrical conductivity, preventing CoTe agglomeration, and accommodating structural damage caused by CoTe's volume changes during charge‐discharge cycling. Simultaneously, the anchored CoTe nanoparticles reduce the surface energy of Ti_3_C_2_ MXene nanosheets, effectively preventing them from restacking. As a result of the synergistic effect, the composite CoTe@Ti_3_C_2_ anode demonstrates impressive cycling performance, delivering a capacity of 698 mAh g^−1^ after 1000 cycles at 0.1 A g^−1^ and maintaining long‐term cycling stability over 1300 cycles at a high current density of 1 A g^−1^. Moreover, battery tests confirmed the superior electrochemical performance of the CoTe@Ti_3_C_2_ composite electrode, outperforming pristine CoTe and Ti_3_C_2_ electrodes. Additionally, synchrotron in situ X‐ray diffraction (XRD) and in situ X‐ray absorption spectroscopy (XAS) were used to analyze the charge storage mechanisms of the CoTe@Ti_3_C_2_ electrode material during charge‐discharge tests. Overall, the CoTe@Ti_3_C_2_ composite electrode exhibits outstanding electrochemical performance, proving its potential as a high‐performance anode material for LIB applications.

## 2 Results and Discussion

**Figure 1:**
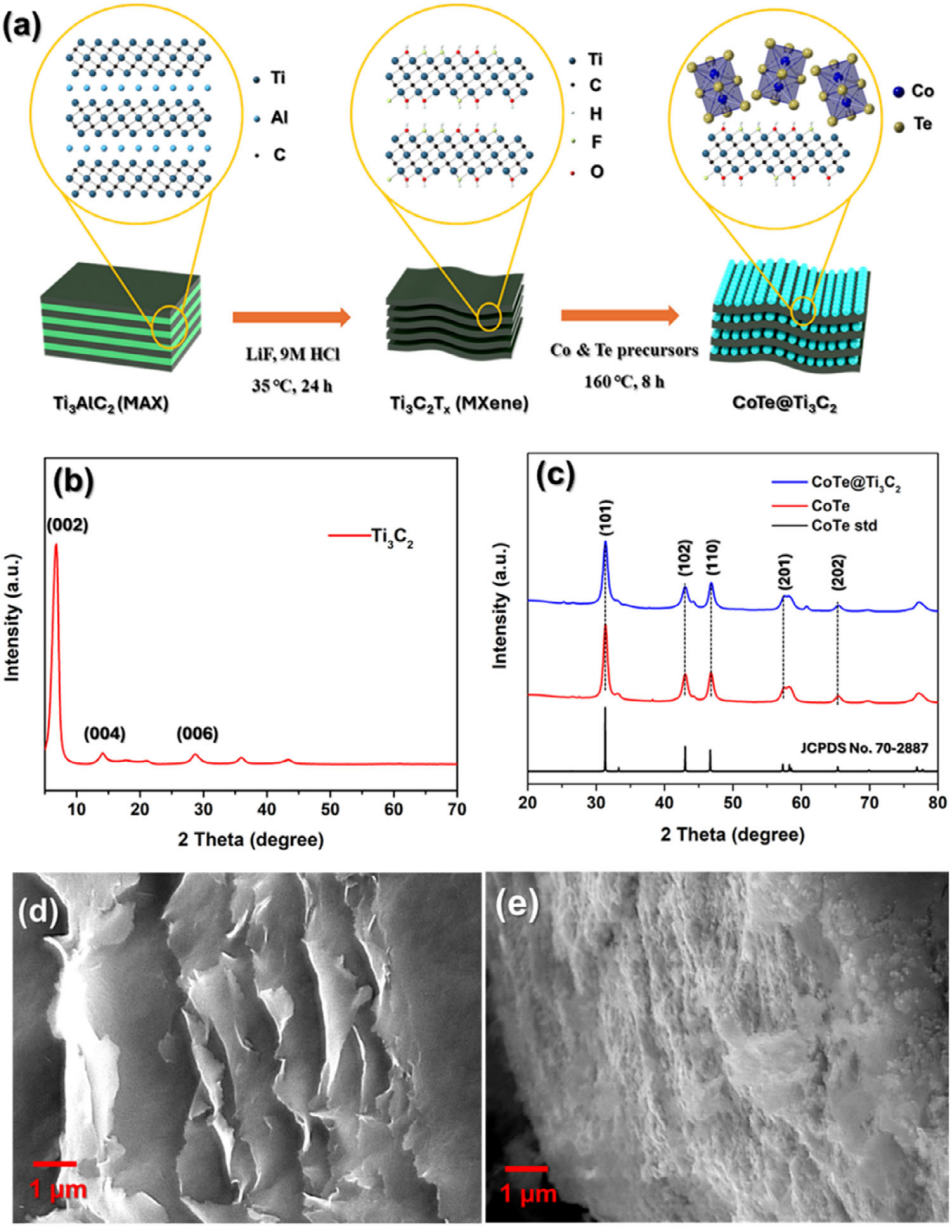
Structural characterization of Ti_3_C_2_ MXene, CoTe, and CoTe@Ti_3_C_2_. a) Schematic illustrating the design and synthesis of CoTe@Ti_3_C_2_ composites. b,c) XRD patterns of b) Ti_3_C_2_ and c) CoTe and CoTe@Ti_3_C_2_. d,e) FESEM images of d) Ti_3_C_2_ and e) CoTe@Ti_3_C_2_.

The design and synthesis of CoTe@Ti_3_C_2_ composite is schematically illustrated in **Figure** **1**a. Briefly, Ti_3_C_2_ MXene nanosheets were prepared by etching the Al layer from Ti_3_AlC_2_ MAX phase, followed by exfoliation of the multilayers via ultrasonication (see the experimental section for details). To synthesize CoTe@Ti_3_C_2_ composites, Co and Te precursors were mixed with Ti_3_C_2_ MXene nanosheets in an autoclave and heated at 160 °C for 8 h, facilitating the growth of CoTe nanoparticles on the MXene nanosheets. Figure 1b–e illustrates the structural characterization of Ti_3_C_2_ MXene, CoTe, and CoTe@Ti_3_C_2_. The crystal structures of the synthesized materials were confirmed by XRD measurements. The XRD pattern of Ti_3_C_2_ MXene nanosheets (Figure 1b) exhibits a characteristic (002) peak, confirming its structural integrity.^[^ ^63^ ^]^ The XRD patterns of CoTe and CoTe@Ti_3_C_2_ (Figure 1c) display similar peaks at 31.3°, 43.0°, 46.7°, 57.3°, and 61.2°, corresponding to the (101), (102), (110), (201), and (202) planes. These peaks are consistent with the standard hexagonal phase of CoTe (JCPDS PDF #70‐2887). Notably, the disappearance of (002) peak of Ti_3_C_2_ MXene in the CoTe@Ti_3_C_2_ pattern, likely due to reduced diffraction intensity caused by structural hybridization. Figure S1 (Supporting Information) presents the XRD Rietveld refinement of CoTe and CoTe@Ti_3_C_2_, further confirming the phase composition and crystalline quality of the composite. The high‐resolution field emission scanning electron microscope (FESEM) image of Ti_3_C_2_ MXene (Figure 1d) reveals a well‐separated, layered structure. Figure S2 (Supporting Information) presents the high‐resolution FESEM image of the synthesized CoTe nanoparticles, showing noticeable agglomeration. In contrast, after the incorporation of CoTe into Ti_3_C_2_ MXene, CoTe nanoparticles covered the surface of the Ti_3_C_2_ MXene nanosheets without any agglomeration, as shown in Figure 1e.

**Figure 2:**
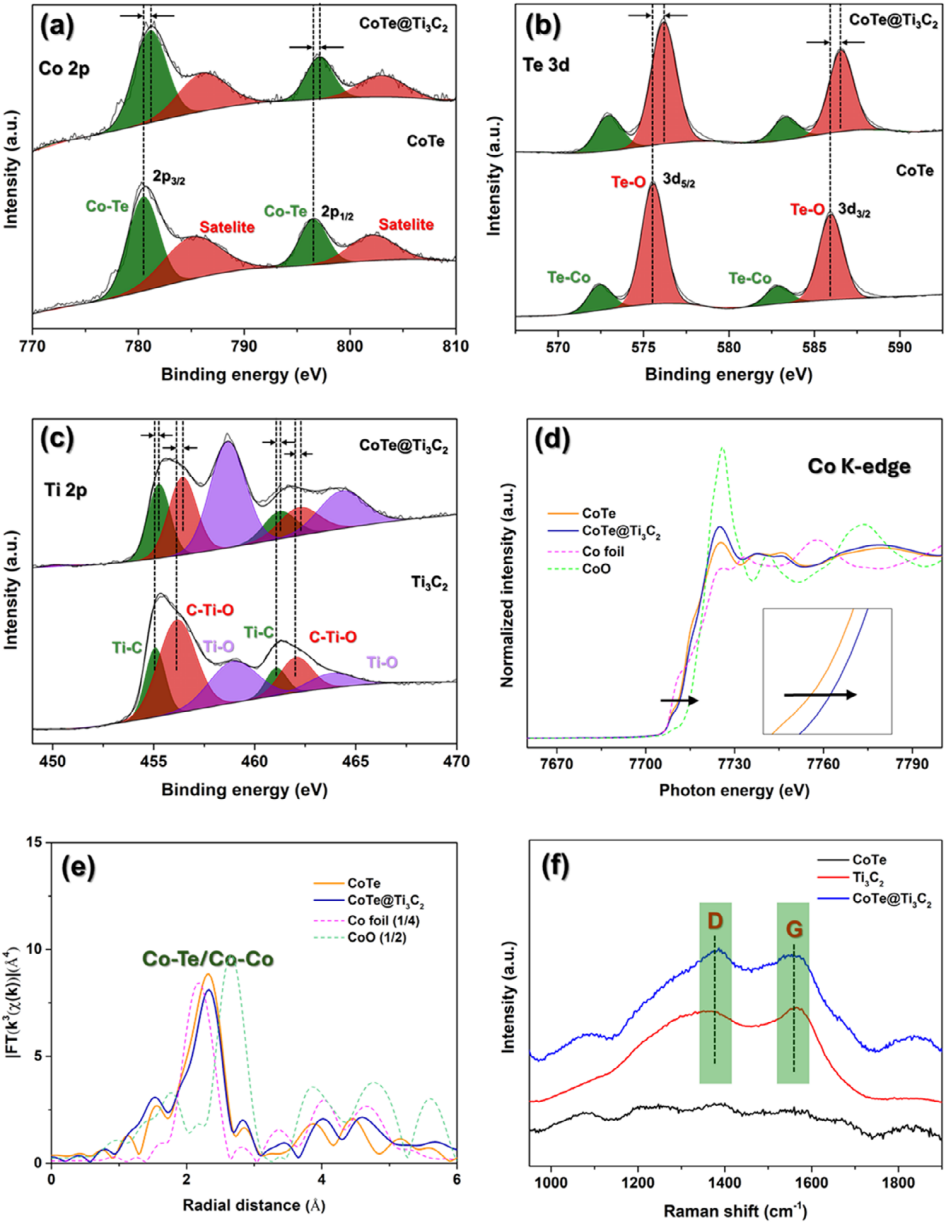
a–c) XPS spectra of Ti_3_C_2_ MXene, CoTe, and CoTe@Ti_3_C_2_: (a) Co 2p, (b) Te 3d, and (c) Ti 2p. d,e) XAS profiles of CoTe and CoTe@Ti_3_C_2_: (d) Co K‐edge XANES and (e) RDF of K^3^‐weighted Co K‐edge EXAFS spectra without phase shift correction. f) Raman spectra of Ti_3_C_2_ MXene, CoTe and CoTe@Ti_3_C_2_. The intensity values for Co foil and CoO in (e) are scaled by factors of 0.25 and 0.5, respectively.

X‐ray photoelectron spectroscopy (XPS) was employed to investigate the surface compositions, valence state of different elements, and the interactions between CoTe and Ti_3_C_2_ MXene. Figure S3 (Supporting Information) presents the survey spectra of Ti_3_C_2_, CoTe, and CoTe@Ti_3_C_2_. The survey spectrum of CoTe@Ti_3_C_2_ confirms the presence of Co, Te, O, N, and Ti components, indicating the formation of a composite structure between CoTe and Ti_3_C_2_ MXene. The high‐resolution core‐level Co 2p XPS spectrum of CoTe shows two prominent peaks at 780.50 and 796.33 eV, corresponding to Co 2p_3/2_ and Co 2p_1/2_, respectively, along with satellite peaks at 785.3 and 802.1 eV (**Figure** **2**a).^[^ ^64^ ^]^ Similarly, the core‐level Te 3d XPS spectrum of CoTe (Figure 2b) reveals two distinct peaks at 575.6 and 586.0 eV, assigned to Te 3d_5/2_ and Te 3d_3/2_, respectively, accompanied by satellite peaks at 572.4 and 582.9 eV.^[^ ^30^, ^65^ ^]^ Compared to CoTe, the deconvoluted Co 2p and Te 3d peaks of CoTe@Ti_3_C_2_ exhibit an upward shift, indicating an increase in the valence state. This shift indicates an interaction between CoTe and Ti_3_C_2_ MXene. Furthermore, as shown in Figure 2c, the appearance of Ti 2p peaks in CoTe@Ti_3_C_2_ further confirms the formation of a composite structure.

To further investigate the electronic and local atomic structures of CoTe and CoTe@Ti_3_C_2_, element‐specific XAS was conducted. Figure 2d,e presents the Co K‐edge X‐ray absorption near‐edge structure (XANES) spectra and the radial distribution function (RDF) of Fourier transformed (FT) *k* ^3^‐weighted Co K‐edge extended X‐ray absorption fine structure (EXAFS) spectra without phase shift correction, respectively. Reference spectra of Co and CoO are included for comparison. The normalized XANES spectra (Figure 2d) show a redshift in the absorption edge of CoTe@Ti_3_C_2_ compared to CoTe, indicating an increased Co valence state after the introduction of Ti_3_C_2_ MXene. EXAFS spectra (Figure 2e) exhibit a prominent peak corresponding to the single scattering of Co─Te, with minimal Co─Co contributions. Figure S4 (Supporting Information) presents the radial distances of different atoms from the central Co atom in the CoTe crystal structure. The reduced intensity of the Co─Te/Co─Co peak in CoTe@Ti_3_C_2_ further confirms the increase in the Co valence. These XAS results align with the findings from the XPS analysis, further validating the interaction between CoTe and Ti_3_C_2_ MXene.

Raman spectroscopy was employed to evaluate the degree of graphitization in Ti_3_C_2_, CoTe, and CoTe@Ti_3_C_2_. The Raman spectra (Figure 2f) show two characteristic peaks at 1384 and 1564 cm^−1^, corresponding to the disordered sp^3^ carbon (D band) and graphitic sp^2^ structure (G band), respectively.^[^ ^66^ ^]^ Compared to CoTe, the presence of D and G bands in CoTe@Ti_3_C_2_ confirms the hybridization between CoTe and MXene. After the peak fitting (Figure S5, Supporting Information), the calculated intensity ratios of the D and G bands (I_D_/I_G_) for Ti_3_C_2_ and CoTe@Ti_3_C_2_ are 0.9 and 1.1, respectively. The higher I_D_/I_G_ ratio in CoTe@Ti_3_C_2_ indicates a greater number of defects and active sites, which enhance charge transfer and improve electrochemical performance.

**Figure 3:**
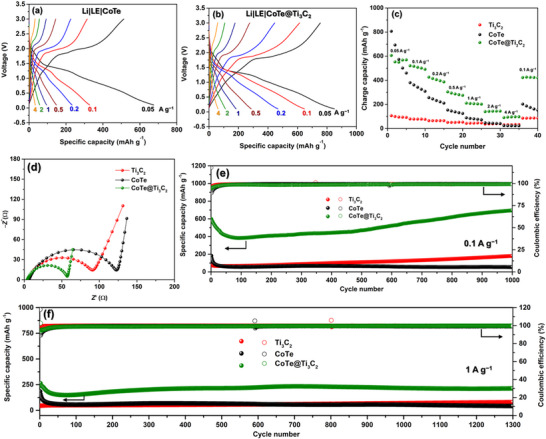
Battery performances: a) Charge‐discharge profiles of Li|LE|CoTe cell at different current densities. b) Charge‐discharge profiles of Li|LE|CoTe@Ti_3_C_2_ cell at different current densities. c) Rate performance of batteries using Ti_3_C_2_, CoTe, and CoTe@Ti_3_C_2_ electrodes. d) EIS spectra of batteries employing Ti_3_C_2_, CoTe, and CoTe@Ti_3_C_2_ electrodes after charge‐discharge test. e) Cycling performance of batteries with Ti_3_C_2_, CoTe, and CoTe@Ti_3_C_2_ electrodes at a current density 0.1 A g^−1^. f) Cycling performance of batteries with Ti_3_C_2_, CoTe, and CoTe@Ti_3_C_2_ electrodes at a current density 1 A g^−1^.

**Figure** **3**a,b shows the charge‐discharge performance of Li|LE|CoTe and Li|LE|CoTe@Ti_3_C_2_ batteries at 30  °C. The batteries were tested at different current densities, ranging from 0.05 to 4 A g^−1^, within a voltage range of 0.1–3.0 V. Compared to the Li|LE|CoTe battery (Figure 3a), the Li|LE|CoTe@Ti_3_C_2_ battery demonstrates higher capacities and lower charge transfer resistance during charge‐discharge performance at all current densities (Figure 3b). Figure 3c exhibits the rate capabilities of batteries with different electrodes. The Li|LE|CoTe@Ti_3_C_2_ battery maintains stable capacities at all current densities and effectively recovers its original capacity when the current density returns to 0.1 A g^−1^. In contrast, the batteries with Ti_3_C_2_ and CoTe electrodes exhibit capacity fading and fail to recover their capacity when the current density returns to 0.1 A g^−1^. Figure 3d presents the electrochemical impedance spectroscopy (EIS) profiles of batteries with Ti_3_C_2_, CoTe, and CoTe@Ti_3_C_2_ electrodes. The CoTe@Ti_3_C_2_ electrode exhibits the lowest total impedance, indicating enhanced charge transfer kinetics compared to Ti_3_C_2_ and CoTe electrodes.

To evaluate the cycling performance, batteries with Ti_3_C_2_, CoTe, and CoTe@Ti_3_C_2_ electrodes were tested at a current density of 0.1 A g^−1^ (Figure 3e). The battery with the Ti_3_C_2_ MXene electrode exhibits poor cycling performance, which can be attributed to the restacking of MXene nanosheets that hinders the Li^+^ transfer within the electrode.^[^ ^61^, ^62^ ^]^ In contrast, the battery with the CoTe electrode delivers a high initial capacity, but suffers severe capacity loss, losing 80% of its initial capacity within 50 cycles of charge‐discharge. This can be attributed to the gradual structural degradation (i.e., particle pulverization, cracking, and loss of electrical contact between active materials and the conductive network) of CoTe caused by volume changes during cycling.^[^ ^67^, ^68^, ^69^, ^70^ ^]^ In comparison with the cycling performances of Ti_3_C_2_ and CoTe electrodes, the battery with the CoTe@Ti_3_C_2_ electrode demonstrates superior cycling performance, delivering a capacity of 698 mAh g^−1^ after 1000 cycles at 0.1 A g^−1^. This improvement is attributed to the incorporation of MXene into the CoTe structure, which stabilizes the CoTe during cycling and prevents structural destruction. However, the specific capacity of the CoTe@Ti_3_C_2_ electrode exhibits an initial decline and stabilizes after ≈50 cycles. To investigate this phenomenon, we analyzed the EIS evolution during cycles (Figure S6, Supporting Information). The initial capacity loss observed within the first ≈50 cycles is mainly due to irreversible processes such as solid electrolyte interphase (SEI) formation and incomplete conversion during the early cycles. EIS reveals a progressive decrease in R_ct_ up to the 50th cycle, suggesting improved interfacial kinetics and stabilization of the SEI layer. This correlates with the subsequent capacity stabilization, indicating that the CoTe@Ti_3_C_2_ architecture effectively accommodates volume changes and enhances Li^+^ transport following the initial activation period. In addition, the gradual increase in capacity after 500 cycles is attributed to the contribution from Ti_3_C_2_. A similar trend is observed in the cycling profile of Ti_3_C_2_ alone. This phenomenon is commonly reported in carbon‐based composites and normally attributed to improved Li^+^ diffusion kinetics resulting from a gradual activation process and reversible interactions between active materials and the electrolyte.^[^ ^71^, ^72^, ^73^ ^]^ Figure S7 (Supporting Information) presents the cycling performances of CoTe@Ti_3_C_2_ electrodes with different MXene contents. The results show that an optimized Ti_3_C_2_ content (≈20 wt.%) provides a balance between mechanical support, electrical conductivity, and active material availability. Consequently, CoTe@Ti_3_C_2_ with 20wt.% Ti_3_C_2_ was selected for further in situ analysis. Additionally, the cycling performance of batteries was tested at a higher current density of 1 A g^−1^ using Ti_3_C_2_, CoTe, and CoTe@Ti_3_C_2_ electrodes. As shown in Figure 3f, the CoTe@Ti_3_C_2_ electrode demonstrates superior cycling stability over 1300 cycles compared to the cycling performances of Ti_3_C_2_ and CoTe electrodes. Table S1 (Supporting Information) presents the comparison of the electrochemical performance of CoTe@Ti_3_C_2_ with other recently reported anode materials. As shown in Table S1 (Supporting Information), our CoTe@Ti_3_C_2_ demonstrates superior long‐term cycling stability.

Based on the above results, CoTe@Ti_3_C_2_ composite exhibits excellent electrochemical performance. The electrochemical kinetics of the as prepared CoTe@Ti_3_C_2_ were studied using cyclic voltammetry (CV), galvanostatic intermittent titration technique (GITT), and EIS. Figure S8a (Supporting Information) presents the CV at different scan rates (0.3 to 1.2 mV s^−1^). The relationship between peak current (*i*) and scan rate (*v*) is described by the following equation:

(1) $$\log\left(i\right)=\log\left(a\right)+b\;\log\left(v\right)$$

here, a and b are constants. When b = 1, it implies surface‐controlled pseudocapacitive process, while b = 0.5 implies diffusion‐controlled behavior of the charge storage mechanism. As shown in Figure S8b,c (Supporting Information), the calculated b values of cathodic and anodic peaks are 0.90 and 0.85, respectively, indicating that the charge storage in CoTe@Ti_3_C_2_ is dominated by pseudocapacitive‐controlled behavior. The pseudocapacitive contribution can be further calculated by the following equation:

(2) $$i\;=k_{1}v+k_{2}v^{1/2}$$

where, *k*_1_ *v* represents pseudocapacitive contribution, and *k*_2_ *v* ^1/2^ accounts for diffusion‐controlled contribution. As shown in Figure S8d (Supporting Information), the pseudocapacitive contribution at a scan rate of 1.2 mV s^−1^ is 87.4%. Figure S9a (Supporting Information) presents the GITT profile of CoTe@Ti_3_C_2_ during the first discharge and charge processes, along with the calculated diffusion coefficients (Figure S9b,c, Supporting Information). The average Li^+^ diffusion coefficient during the first discharge and charge steps were 3.1 × 10^−12^ and 5.2 × 10^−12^ cm^2^ s^−1^, respectively. To further confirm the diffusion coefficient, EIS measurement was used. Figure S9d (Supporting Information) shows the Nyquist EIS profile CoTe@Ti_3_C_2_ cell along with a fitted curve based on the equivalent circuit model (inset). The calculated Li^+^ diffusion coefficient, derived from the fitted parameters, is 2.71 × 10^−12^ cm^2^ s^−1^. These values are consistent with the previously reported data, confirming the rapid Li^+^ diffusion kinetics of the CoTe@Ti_3_C_2_ composite.^[^ ^74^ ^]^

**Figure 4:**
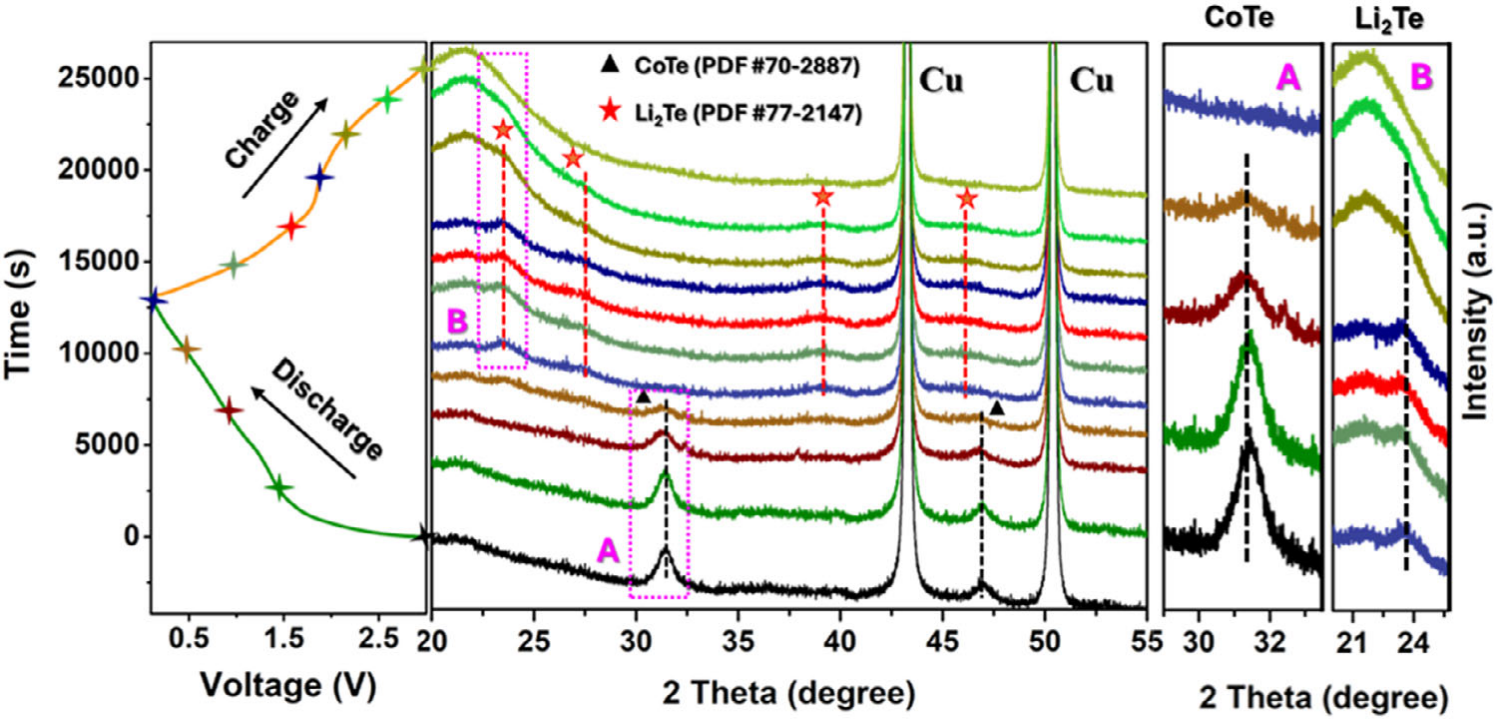
In situ synchrotron XRD patterns for the Li|LE|CoTe@Ti_3_C_2_ cell during the initial charge‐discharge cycle at 0.1 mA g^−1^. The horizontal axis corresponds to specific 2‐theta regions, while the vertical axis represents time. The corresponding voltage curve is shown on the left. Enlarged areas (A,B) highlight key regions: A) 29– 33.3°, focusing on the primary peaks of CoTe, and B) 20–25.6°, focusing on the primary peaks of Li₂Te.

To investigate the structural behavior of the CoTe@Ti_3_C_2_ electrode during the electrochemical charge‐discharge process, in situ XRD was performed on a Li|LE|CoTe@Ti_3_C_2_ cell. **Figure** **4** presents the in situ diffraction patterns recorded during the initial discharge and charge step for the Li|LE|CoTe@Ti_3_C_2_ cell operating at 0.1 mA g^−1^ within a voltage range of 0.1–3.0 V. As shown in Figure 4, two well‐defined and strong peaks at 43.4° and 50.5° are attributed to Cu current collectors. At the beginning of the discharge process, the XRD pattern exhibits two peaks at 31.3° and 46.7°, corresponding to the (101) and (110) planes of hexagonal CoTe. Upon discharge to 0.1 V, the CoTe peaks disappear, and new peaks associated with Li_2_Te emerge, indicating the conversion of CoTe into Li_2_Te and an amorphous Co phase.^[^ ^74^ ^]^ The absence of Co peaks in the XRD pattern confirms the amorphous phase of Co (Figure 6a). For clarity, a closer look at the 2 theta ranges 29–33.3° (enlarged area A) and 20–25.6° (enlarged area B) focuses on the primary peaks of CoTe and Li_2_Te, respectively. Upon charging to 3.0 V, the peaks of Li_2_Te disappeared. Notably, at the end of the first cycle, the absence of CoTe peaks confirms the amorphization of CoTe@Ti_3_C_2_. Overall, the in situ XRD analysis provides insights into the structural changes of the CoTe@Ti_3_C_2_ electrode during the charge‐discharge process.

The possible charge storage mechanism can be described by the following equations:^[^ ^41^, ^74^ ^]^

Discharge process:

(3) $$CoTe+2Li^{+}+2e^{-}\to Li_{2}Te+Co\left(amorphous\right)$$

Charge process:

(4) $$Li_{2}Te+Co\left(amorphous\right)\to CoTe\left(amorphous\right)+2Li^{+}+2e^{-}$$

**Figure 5:**
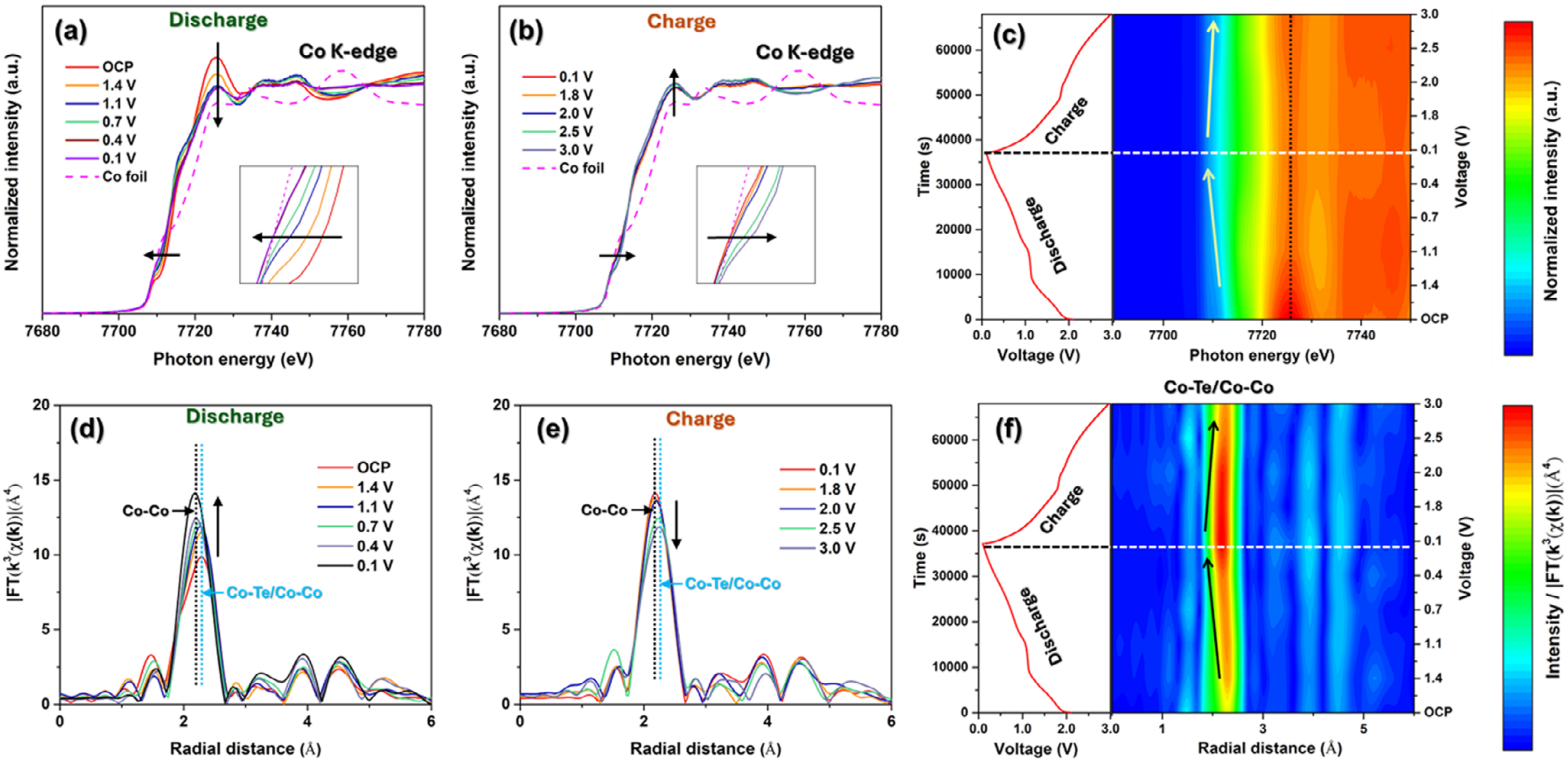
a,b) Co K‐edge XANES profiles of Li|LE|CoTe@Ti_3_C_2_ battery during (a) discharge and (b) charge at the current density of 0.05 A g^−1^. c) Contour map of Co K‐edge XANES for the in situ Li|LE|CoTe@Ti_3_C_2_ battery during first charge‐discharge cycle at 0.05 A g^−1^. d,e) RDF of k^3^‐weighted Co K‐edge EXAFS spectra without phase shift correction of Li|LE|CoTe@Ti_3_C_2_ battery during (d) discharge and (e) charge at 0.05 mA g^−1^. f) Contour map of RDF of k^3^‐weighted Co K‐edge EXAFS spectra without phase shift correction for the in situ Li|LE|CoTe@Ti_3_C_2_ battery during the first charge‐discharge cycle at 0.05 mA g^−1^.

To investigate the variations of the Co valence and local structure in the Li|LE|CoTe@Ti_3_C_2_ cell during Li^+^ insertion/extraction, in situ XAS measurements were conducted. **Figure** **5**a,b presents the in situ Co K‐edge XANES spectra recorded during the discharge and charge steps, respectively, for the Li|LE|CoTe@Ti_3_C_2_ cell operating at 0.05 A g^−1^_._ The corresponding contour map, with different charge and discharge positions indicated in the voltage profile, is shown in Figure 5c. During discharge to 0.1 V (Figure 5a), the normalized Co K‐edge XANES spectra exhibited a significant blueshift in the absorption edge between the OCP and the 0.1 V discharged state, indicating a decrease in the Co valence. Additionally, the corresponding intensity of the white line decreased during the discharge process, resulting in the XANES spectrum trending toward the behavior of Co foil. This result indicates the conversion of CoTe to Li_2_Te and an amorphous Co during the discharge process. Upon charging (0.1 to 3.0 V), the normalized Co K‐edge XANES spectra showed a redshift in the absorption edge, with the 3.0 V spectrum nearly returning to its initial state, indicating an increase in the Co valence state and the formation of amorphous CoTe@Ti_3_C_2_ (Figure 5b). The contour map (Figure 5c) illustrates the shift in the absorption edge and changes in the white line peak intensity, as marked by the solid light‐yellow and black dotted lines, respectively, during the charge‐discharge step.

**Figure 6:**
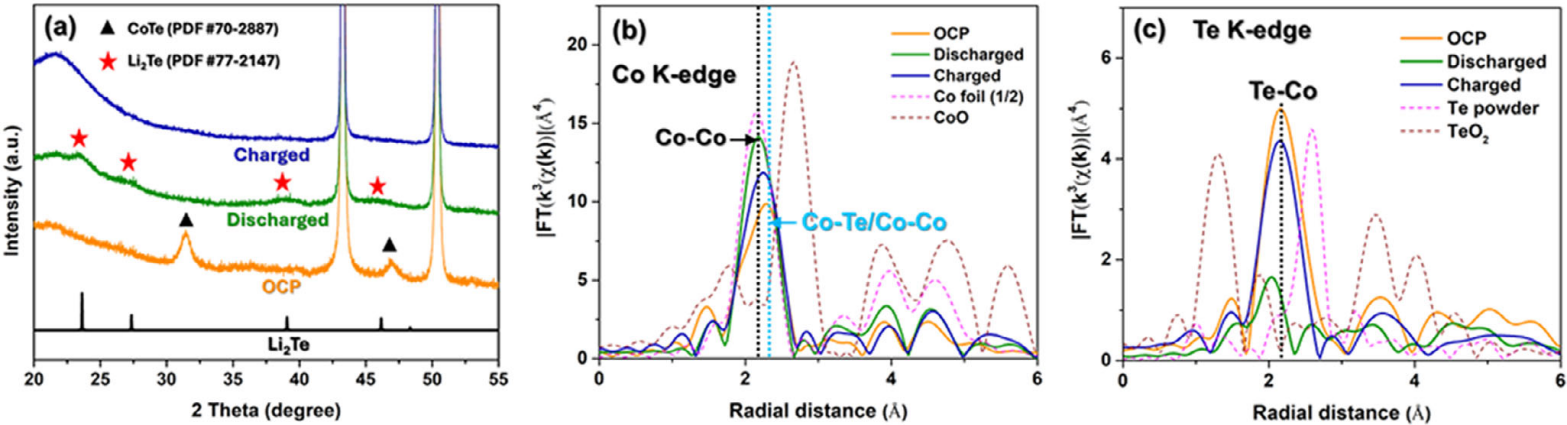
a) XRD patterns of the CoTe@Ti_3_C_2_ electrode at different stages of charge‐discharge cycle. b) RDF of k^3^‐weighted Co K‐edge EXAFS spectra without phase shift correction for the CoTe@Ti_3_C_2_ electrode at different stages of charge‐discharge cycle. c) RDF of k^3^‐weighted Te K‐edge EXAFS spectra without phase shift correction for the CoTe@Ti_3_C_2_ electrode at different stages of charge‐discharge cycle. The intensity of Co foil in (b) is scaled by a factor of 0.5.

Figure 5d,e presents the in situ Co K‐edge EXAFS spectra recorded during the discharge and charge cycles, respectively, for the Li|LE|CoTe@Ti_3_C_2_ cell operating at 0.05 A g^−1^. The corresponding contour map is shown in Figure 5f with different charge and discharge positions indicated in the voltage profile. Figure 5d shows a well‐resolved prominent peak attributed to the single scattering of Co─Te/Co─Co coordination shell within the CoTe crystal structure. Upon discharging to 0.1 V, the peak shifts to a lower bond distance with increased intensity. At the end of the discharge process, the RDF of the *k* ^3^‐weighted Co K‐edge EXAFS peak aligns with the Co─Co peak of the Co foil (**Figure** **6**b), indicating the conversion of CoTe into amorphous Co.^[^ ^40^ ^]^ Figure S10 (Supporting Information) compares the radial distances of different atoms from the central Co atom in (top) the CoTe crystal structure and (bottom) the Co crystal structure. The shorter Co─Co bond distance in the Co crystal structure, compared to Co─Co in CoTe, further confirms this conversion. At the end of the charge process (3.0 V), both the peak position and intensity nearly return to their initial position, confirming the formation of amorphous CoTe@Ti_3_C_2_ (Figures 5e and 6b). The contour map in Figure 5f highlights the changes in the CoTe@Ti_3_C_2_ peak position and intensity, as indicated by the solid black lines, during charge‐discharge process.

To further investigate the CoTe@Ti_3_C_2_ evolution during the charge‐discharge process, in situ Te K‐edge EXAFS spectra were also measured (Figure 6c), with the corresponding Te K‐edge XANES spectra presented in Figure S11 (Supporting Information). In the RDF of *k* ^3^‐weighted Te K‐edge EXAFS spectra, the first coordination shell corresponds to Te─Co bonding, consistent with the Co─Te bond observed in the Co K‐edge EXAFS spectra (Figure 6b). Upon full discharge (0.1 V), the amplitude related to the first coordination shell decreases significantly, indicating the replacement of Co in the Te─Co bond by Li, forming Te─Li bonds. This substitution leads to a reduction in spectral intensity and a noticeable shortening of the bond distance due to the lighter mass and smaller ionic radius of Li. At the end of the charge process (3.0 V), a partial recovery of the Te─Co shell is observed. However, discrepancies from the initial state due to structural disorder induced by the Te─Li to Te─Co reconversion. This is further corroborated by in situ XRD, where the charged spectrum indicates an amorphous phase. The consistency between the XRD and EXAFS results further validates the structural evolution of the CoTe@Ti_3_C_2_ electrode during charge‐discharge cycling.

Motivated by the outstanding performance of CoTe@Ti_3_C_2_ in half‐cell configurations, a full cell was assembled using LiFePO_4_ as the cathode material. The pre‐treated CoTe@Ti_3_C_2_ served as the anode, paired with a LiFePO_4_ cathode in the full cell, as shown in Figure S12a (Supporting Information). The full cell displayed an excellent rate performance at different current densities, which increased from 0.05 to 5 C (Figure S12b, Supporting Information). Notably, when the current rate returned to 0.1 C, the specific capacity was recovered, indicating an excellent rate performance of the full cell. These results further demonstrated that the CoTe@Ti_3_C_2_ could be applied in full cell applications.

## 3 Conclusion

In this study, we successfully synthesized and characterized a CoTe@Ti_3_C_2_ MXene composite as a high‐performance anode material for LIBs. The integration of CoTe nanoparticles with Ti_3_C_2_ MXene significantly enhanced the electrical conductivity, structural stability, and electrochemical performance of the CoTe@Ti_3_C_2_ electrode. The synergistic interaction between CoTe and Ti_3_C_2_ MXene effectively minimized CoTe agglomeration, accommodated its volume change during charge‐discharge cycling, and reduced MXene nanosheet restacking, addressing key challenges associated with both materials. As a result, the CoTe@Ti_3_C_2_ electrode exhibited outstanding cycling stability, achieving 698 mAh g^−1^ after 1000 cycles at 0.1 mA g^−1^, a more than tenfold increase compared to CoTe, while also demonstrating long‐term cycling stability over 1300 cycles at 1 mA g^−1^. In situ synchrotron XRD and XAS analyses provided critical insights into the charge storage mechanisms, highlighting the potential of CoTe@Ti_3_C_2_ for practical LIB applications. Overall, this study features CoTe@Ti_3_C_2_ composites as a promising candidate for next‐generation LIB technology, offering a viable route toward high‐capacity and stable energy storage.

## 4 Experimental Section

### Materials

Titanium carbide (TiC, 99.5%, 325 mesh, Gredmann), titanium (Ti, 99.8%, >325 mesh, Gredmann), aluminum (Al, 99.7%, >400 mesh, Gredmann), lithium fluoride (LiF; Alfa, USA), hydrochloric acid (HCl; Alfa, USA), cobalt chloride hexahydrate (CoCl_2_.6H_2_O; Sigma, USA), sodium telluride (Na_2_TeO_3_; Sigma, USA), hydrazine hydrate (N_2_H_4_.H_2_O; Sigma, USA), polyvinylidene fluoride (PVdF; Sigma, USA) with an average molecular weight of 534 000 g mol^−1^, and conducting agent (Super‐P; Taiwan Maxwave Co., Taiwan).

### MAX Synthesis

Ti_3_AlC_2_ MAX phase powder was prepared by the conventional solid‐state reaction. TiC, Ti, and Al powders were mixed in a polypropylene jar in a molar ratio of 2:1:1. The mixed powder was subjected to ball milling with ZrO_2_ balls in a weight ratio of 1:2 for 8 h. After ball milling, the homogeneous powder was transferred to an alumina crucible and heated to 1380 °C under 300 SCCM Argon flow for 4 h in a tube furnace with a heating/cooling rate of 3 °C min^−1^. The sintered powder was ground in a mortar and sieved to 400 mesh. The sieved powder was immersed in 9 m HCl for 12 h to remove the impurities, followed by thorough washing with deionized water and drying at 60 °C overnight.

### MXene Synthesis

The few‐layer MXene was synthesized by the MILD method. First, the 1.6 g of LiF (98.5%) was dissolved in 20 mL of 9 m HCl and stirred for 30 min. Subsequently, 1 g of synthesized Ti_3_AlC_2_ was gently added in the solution, and the mixture was stirred at 400 rpm under 35 °C in an oil bath for 24 h. After the etching process, the solution was centrifuged at 3500 rpm to remove the acid supernatant. The sediment was re‐dispersed in deionized water and washed repeatedly until the pH value reached >6. The washed clay‐like sediment was dispersed in N_2_‐saturated deionized water and ultrasonicated for 15 min in an ice bath. Following this step, the solution was centrifuged at 3500 rpm to separate the multi‐layer MXene as sediment, while the dark‐green supernatant containing the few‐layer MXene was retained. The few‐layer MXene dispersion was stored in a refrigerator at a temperature below 4 °C.

### CoTe and CoTe@Ti_3_C_2_ Synthesis

The CoTe and CoTe@Ti_3_C_2_ were synthesized using a hydrothermal method. First, 3 mmol of CoCl_2_·6H_2_O and 3 mmol of Na_2_TeO_3_ were dissolved in 55 mL of deionized water to create a homogeneous mixture. Then, 15 mL of N_2_H_4_.H_2_O was transferred to this mixture and stirred for 30 min. For CoTe@Ti_3_C_2_, different amounts of Ti_3_C_2_ MXene solutions were added at this stage and stirred for an additional 20 min. This mixture was then transferred to a 100 mL Teflon‐lined stainless‐steel autoclave. The autoclave was sealed and heated at 160 °C for 8 h to facilitate the reaction. After the reaction was completed, the resulting sample was collected and washed with deionized water and ethanol using centrifugation. Finally, the collected samples were dried under vacuum at 80 °C for 24 h.

### Material Characterization

The crystal structures of the Ti_3_C_2_, CoTe and CoTe@Ti_3_C_2_ powders were characterized using XRD beamline 01C2 at the 1.5 GeV Taiwan Light Source (TLS) (using wavelength 0.77491 Å) and high‐resolution powder XRD beamline 19A1 at the 3.0 GeV Taiwan Photon Source (TPS) (using wavelength 0.61992 Å) of National Synchrotron Radiation Research Center (NSRRC), Taiwan. The 2‐theta angles of all the obtained XRD spectra were recalculated and converted to corresponding angles for wavelength 1.54 Å. XAS of CoTe and CoTe@Ti_3_C_2_ powders were measured using the Quick scanning‐XAS beamline TPS 44A1 of NSRRC using a transmittance mode. All spectra were collected in ambient conditions. The acquired XAS data was processed using the ATHENA 0.9.26 software. The *k* ^3^‐weighted EXAFS data *χ*(*k*) were Fourier transformed into real (R) space, using a Hanning window (d*k* = 1.0 Å^−1^) in the *k* range of 3.2–12.55 Å. The morphologies of Ti_3_C_2_, CoTe, and CoTe@Ti_3_C_2_ were examined through high‐resolution thermal field emission SEM (JEOL, JSM‐7610F, Japan) with EDS for elemental analysis. XPS analyses were performed on a ULVAC‐PHI PHI 5000 Versaprobe II spectrometer.

### In Situ Synchrotron XRD

In situ XRD measurement for Li|LE|CoTe@Ti_3_C_2_ cell was performed in a transmission mode during electrochemical charge‐discharge test at high‐resolution powder XRD beamline TPS 19A1 at NSRRC. The wavelength of the photon energy used was 0.61992 Å. Modified CR2032 coin cells with a circular opening of 2 mm diameter on both sides were employed for the experiment (Figure S13, Supporting Information). To allow X‐ray transmission, polyimide film was used as windows in the cell. During the experiment, the in situ cell was operated at 0.1 A g^−1^ at room temperature. XRD patterns were continuously collected during the experiment. The 2‐theta angles of the obtained XRD spectra were recalculated and converted to corresponding angles for wavelength 1.54 Å, which is commonly used for XRD analysis.

### In Situ Synchrotron XAS

In situ XAS measurements for a Li|LE|CoTe@Ti_3_C_2_ cell during electrochemical charge‐discharge test were performed at Quick scanning‐XAS beamline TPS 44A1 of NSRRC. Modified CR2032 coin cells (Figure S13, Supporting Information) with circular opening of 5 mm diameter on both sides were employed for the experiment. To allow X‐ray transmission, polyimide film was used as windows in the cell. During the experiment, the in situ cell was operated at 0.05 A g^−1^ at room temperature. The Co K‐edge and Te K‐edge absorption spectra were carried out in a fluorescence mode using multi‐element silicon drift detectors. The acquired XAS data was processed using the ATHENA 0.9.26 software. The *k* ^3^‐weighted EXAFS data *χ*(*k*) were Fourier transformed into real (R) space, using a Hanning windows (d*k* = 1.0 Å^−1^). The *k* range windows were 3.5 to 12.55 Å^−1^ for the Co K‐edge and 3 to 11 Å^−1^ for the Te K‐edge.

### Electrochemical Measurements

For the half cell configuration, an electrode slurry was prepared by mixing 70 wt.% active material (CoTe, Ti_3_C_2,_ and CoTe@Ti_3_C_2_), 20 wt.% conducting agents (super‐P), and 10 wt.% binder (PVDF) with an appropriate amount of NMP as solvent. The mixture was thoroughly mixed to obtain a thick and uniform slurry. Prepared cathode slurries were then evenly coated onto a copper current collector. Subsequently, the coated current collectors were dried at 100 °C under vacuum overnight. During this drying process, the NMP solvent evaporated, leaving behind a dried cathode layer. The dried cathodes were calendared and then cut into circular discs with a diameter of ≈15 mm. The mass loading of the active material in the electrode was 1.7 mg. The CR2032 coin cells were assembled in an Ar‐filled glovebox. Li||CoTe, Ti_3_C_2,_ and CoTe@Ti_3_C_2_ half cells were assembled into CR2032 coin cells using the CoTe, Ti_3_C_2_ and CoTe@Ti_3_C_2_, Li metal, and 1 m LiPF_6_ dissolved in ethylene/dimethyl/diethyl carbonates (EC/DMC/DEC in proportions of 1:1:1 by volume) as the electrolyte. Galvanostatic charge‐discharge tests were conducted for batteries between 0.1 and 3.0 V (vs Li/Li^+^) by using battery test equipment (Neware battery tester, China). CV was conducted using the Autolab workstation at a scan rate of 0.3–1.2 mV s^−1^. The galvanostatic charge‐discharge performance and GITT measurements were carried out on Neware battery tester in a voltage range of 0.1–3.0 V. The GITT was measured with a current pulse of 0.1 A g^−1^ for 420 s followed by a 1200 s of relaxation time to achieve equilibrium potential. The EIS was also conducted on the Autolab workstation in the frequency range of 100 KHz to 1 Hz. For the construction of a full cell, the LiFePO_4_ was utilized as cathode, and CoTe@Ti_3_C_2_ was served as anode with the same electrolyte and separator as in half‐cell. To form stable SEI films, the CoTe@Ti_3_C_2_ anode was first cycled at 0.1 A g^−1^ for 5 cycles between 0.1 and 3.0 V in half cell configuration. LiFePO_4_ mixed with Super P and PVDF (in a weight ratio of 80:10:10) were coated on Al foil. To assure the suitable cathode to anode capacity balance, the mass ratio of LiFePO_4_ to CoTe@Ti_3_C_2_ was maintained at 2:1. The electrochemical performances of CoTe@Ti_3_C_2_||LiFePO_4_ full‐cell were tested by galvanostatic charge‐discharge in the voltage of 0.7–3.3 V. All the mentioned electrochemical measurements were performed at room temperature.

## Conflict of Interest

The authors declare no conflict of interest.

## Supporting information



Supporting Information

## Data Availability

The data that support the findings of this study are available from the corresponding author upon reasonable request.
