# Supplementary material for: Reinforced Capacity and Cycling Stability of CoTe Nanoparticles Anchored on Ti3C2 MXene for Anode Material
Source: Small Methods. 2025 Jul 1;10(2):2500725. doi: 10.1002/smtd.202500725 (PMC12825351; doi:10.1002/smtd.202500725)
Supplement: Supplementary file 1 — Supporting Information [file SMTD-10-2500725-s001.pdf]

Supporting Information

Reinforced capacity and cycling stability of CoTe nanoparticles anchored on Ti_3C_2 MXene for anode material

Ramesh Subramani, Su-Yang Hsu, Wei-Hsiang Huang, Zhiwei Hu, Kueih-Tzu Lu*, Jin-Ming Chen**

R. Subramani, J. M. Chen
Department of Electrophysics, National Yang Ming Chiao Tung University (NYCU), Hsinchu, 300093, Taiwan.
E-mail: jmchen@nycu.edu.tw

S. Y. Hsu, W. H. Huang, K. T. Lu
National Synchrotron Radiation Research Center (NSRRC), Hsinchu, 300092, Taiwan.
E-mail: ktlu@nsrrc.org.tw

Z. Hu
Max Planck Institute for Chemical Physics of Solids, 01187 Dresden, Germany.
E-mail: Zhiwei.Hu@cpfs.mpg.de

R. Subramani and S. Y. Hsu contributed equally to this work.

Keywords: CoTe, Ti_3C_2, MXene, *in-situ* XRD, *in-situ* XAS, LIBs

Supporting Information for:

(1) Rietveld refinement of the XRD patterns for CoTe and CoTe@Ti_3C_2

(2) FESEM image of CoTe

(3) XPS survey spectrum

(4) Radial distances of different atoms from central Co atom in CoTe

(5) Raman spectra of Ti_3C_2, and CoTe@Ti_3C_2

(6 EIS evolution CoTe@Ti_3C_2 during cycling

(7) Cycling performance of batteries

(8) Electrochemical kinetics evaluation of CoTe@Ti_3C_2 through CV

(9) Li^+ diffusion and impedance behavior

(10) Radial distances of different atoms from the central Co atom in CoTe and Co crystal structure

(11) *In-situ* Te K-edge XANES spectra of CoTe@Ti_3C_2 electrode

(12) Full cell configuration and rate performance of the CoTe@Ti_3C_2//$LiFePO_4$

(13) *In-situ* XRD/XAS cell

(14) Comparison of the electrochemical performance of CoTe@Ti_3C_2 with other recently reported anode materials.

1. Rietveld refinement of the XRD patterns for CoTe and CoTe@Ti_3C_2

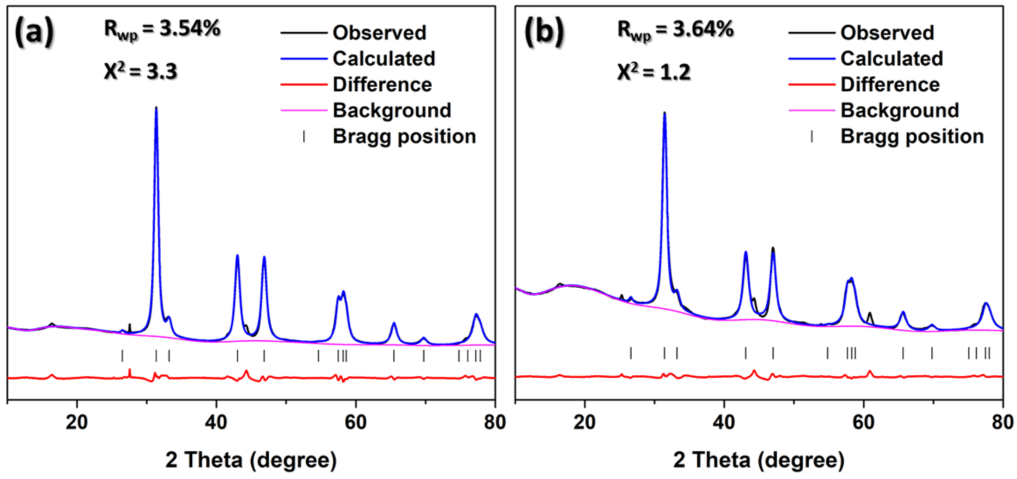

Figure S1 Rietveld refinement of the XRD patterns for (a) CoTe and (b) CoTe@Ti_3C_2.

2. FESEM image of CoTe

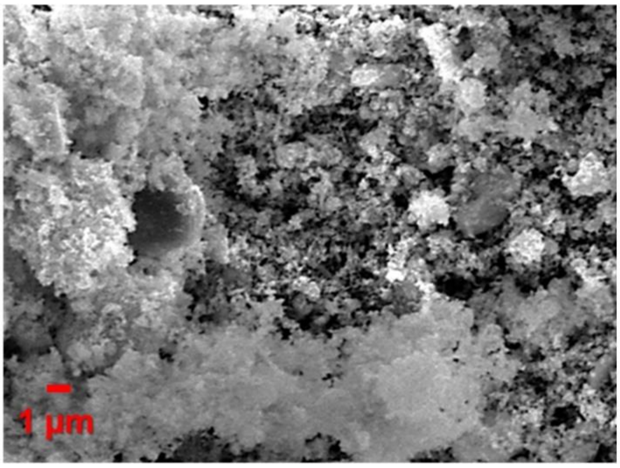

Figure S2 FESEM image of CoTe.

3. XPS survey spectrum

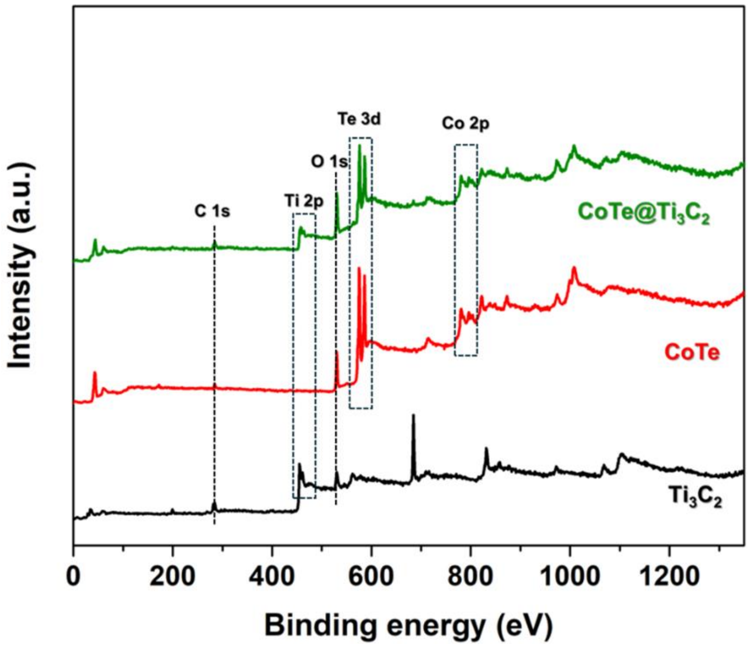

Figure S3 XPS survey spectra of Ti_3C_2, CoTe and CoTe@Ti_3C_2.

4. Radial distances of different atoms from the central Co atom in CoTe

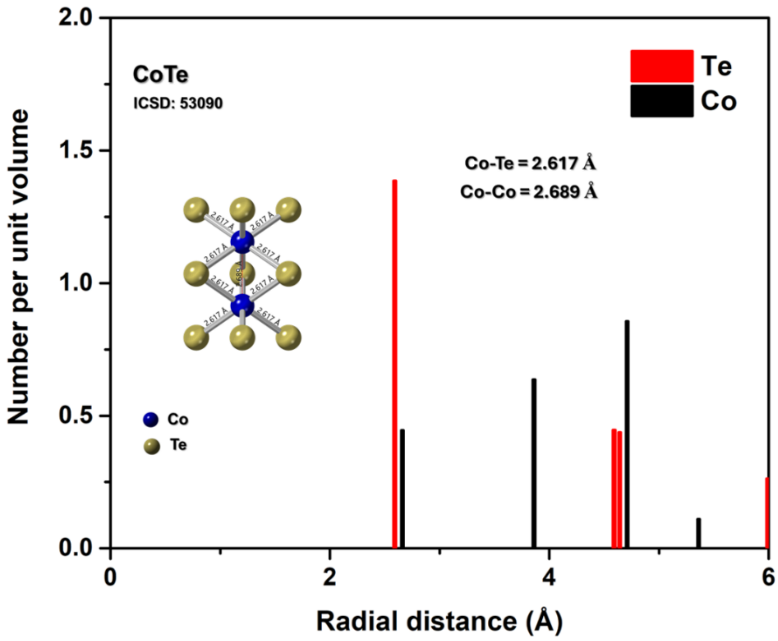

Figure S4 Radial distances of different atoms from the central Co atom in the CoTe crystal structure.

5. Raman spectra of Ti_3C_2, and $CoTe@Ti_3C_2$

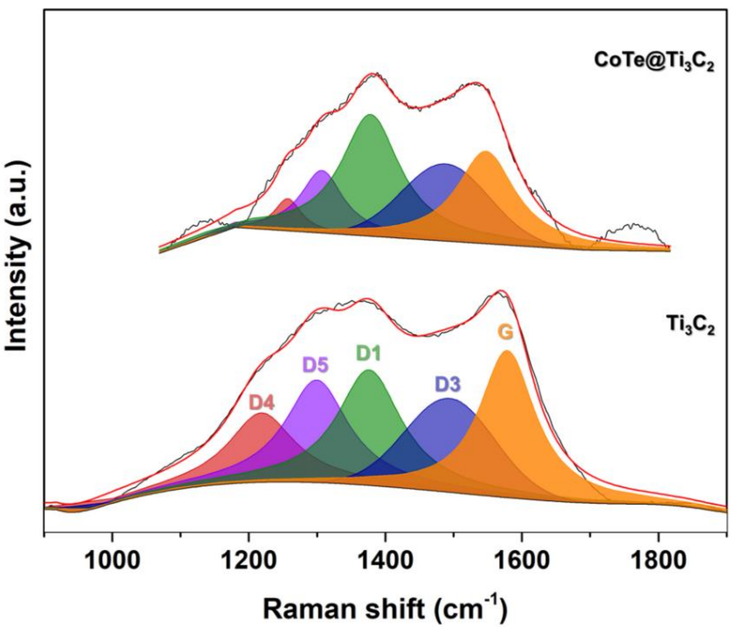

Figure S5 Raman spectra of Ti_3C_2, and $CoTe@Ti_3C_2$.

6. EIS evolution of $CoTe@Ti_3C_2$ during cycling

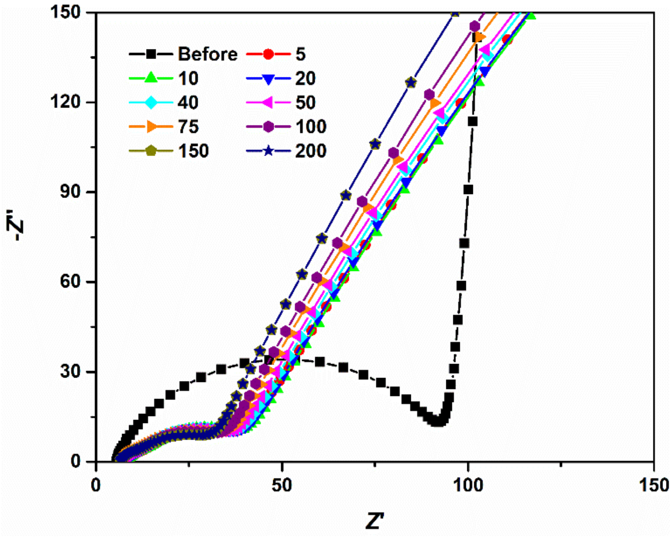

Figure S6 EIS profiles of the $CoTe@Ti_3C_2$ cell before cycling and after 5, 10, 20, 40, 50, 75, 100, 150 and 200 cycles at a current density of 1 A g^{-1}.

7. Cycling performance of batteries

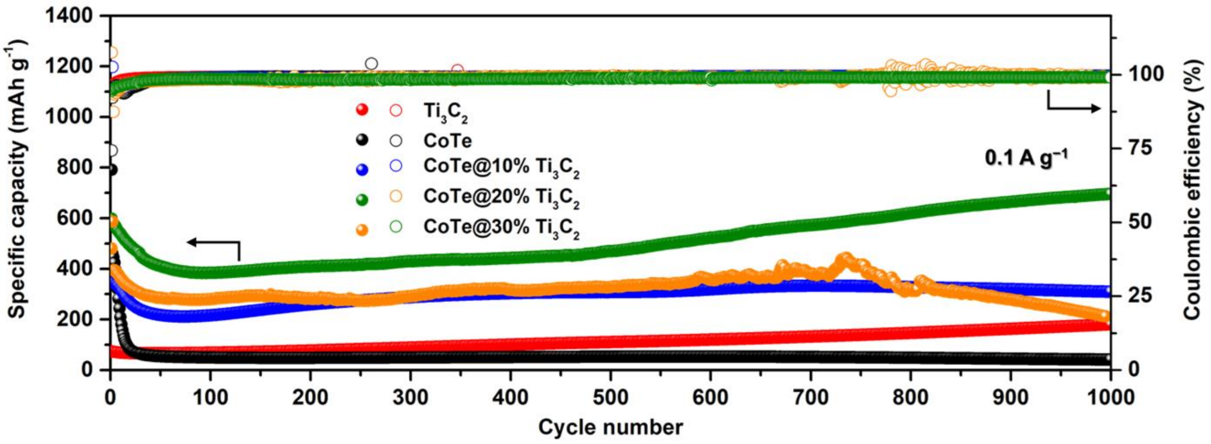

Figure S7 Cycling performance of batteries using Ti_3C_2, CoTe, CoTe@10%Ti_3C_2, CoTe@20%Ti_3C_2, and CoTe@30%Ti_3C_2 electrodes at a current density of 0.1 A g^{-1}.

8. Electrochemical kinetics evaluation of $CoTe@Ti_3C_2$ through CV

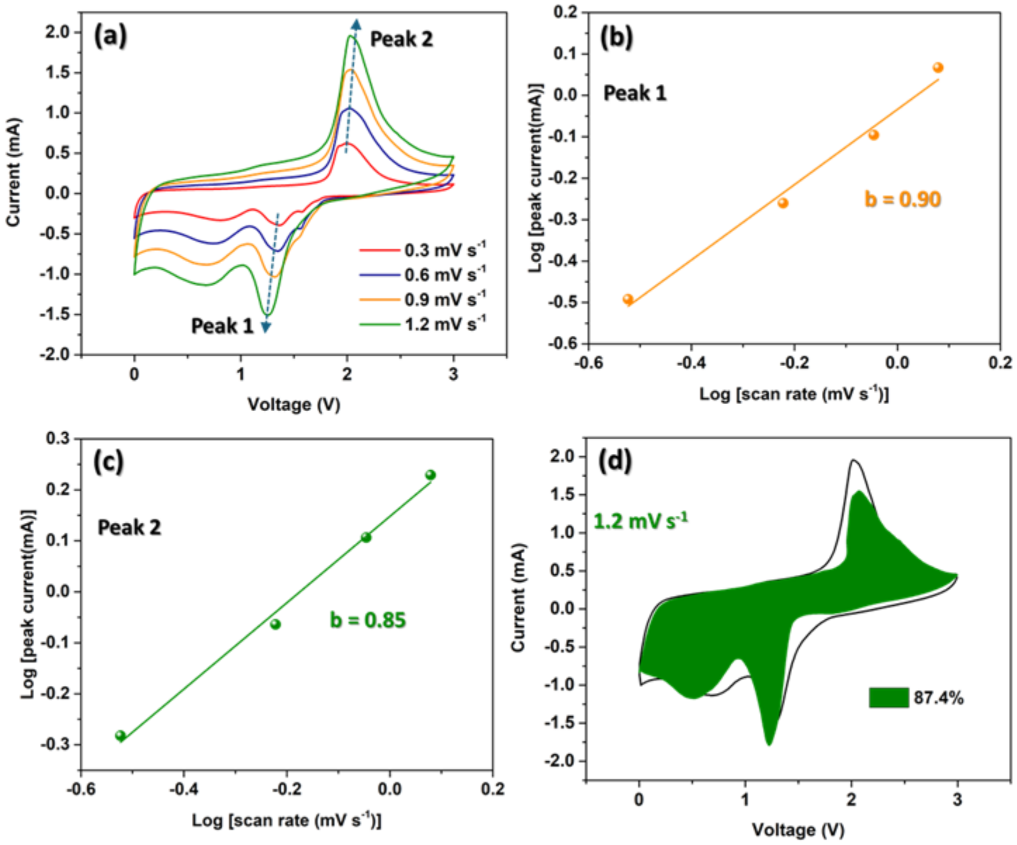

Figure S8 (a) CV profiles of $CoTe@Ti_3C_2$ at different scan rates (0.3-1.2 mV s^{-1}). (b, c) Log (scan rate) vs. log (peak current) plots for $CoTe@Ti_3C_2$, showing (b) cathodic peak (peak 1) and anodic peak (peak 2). (d) Pseudocapacitive contribution (shaded region) of $CoTe@Ti_3C_2$ at a scan rate of 1.2 mV s^{-1}.

9. Li^+ diffusion and impedance behavior

Galvanostatic Intermittent Titration Technique (GITT)

The lithium diffusion coefficients were calculated from the GITT potential profiles using Fick's second law, expressed by the following Equation:

$$D_{Li^+} = \frac{4}{\pi\tau}\left(\frac{\mathrm{m}_B V_M}{M_B S}\right)^2 \left(\frac{\Delta E_s}{\Delta E_\tau}\right)^2$$

Where τ is the duration of the current pulse, m_B and M_B are the active mass and molar mass of active material in the electrode, respectively, V_M is the molar volume, S is the electrode area, ΔE_S is the difference of voltage between initial and steady states, $\Delta E\tau$ is the differences of time between initial and steady states.

The Li^+ diffusion coefficient can be calculated by the EIS results using the following equation:

$$D_{Li^+} = \frac{R^2T^2}{2S^2n^4F^4TC^2\sigma^2}$$

Where *T* is the temperature, *R* is the gas constant, *F* is the Faraday constant, *n* is the number of electrons, *S* is the surface area of the electrode, *C* is the concentration, and σ is the Warburg coefficient.

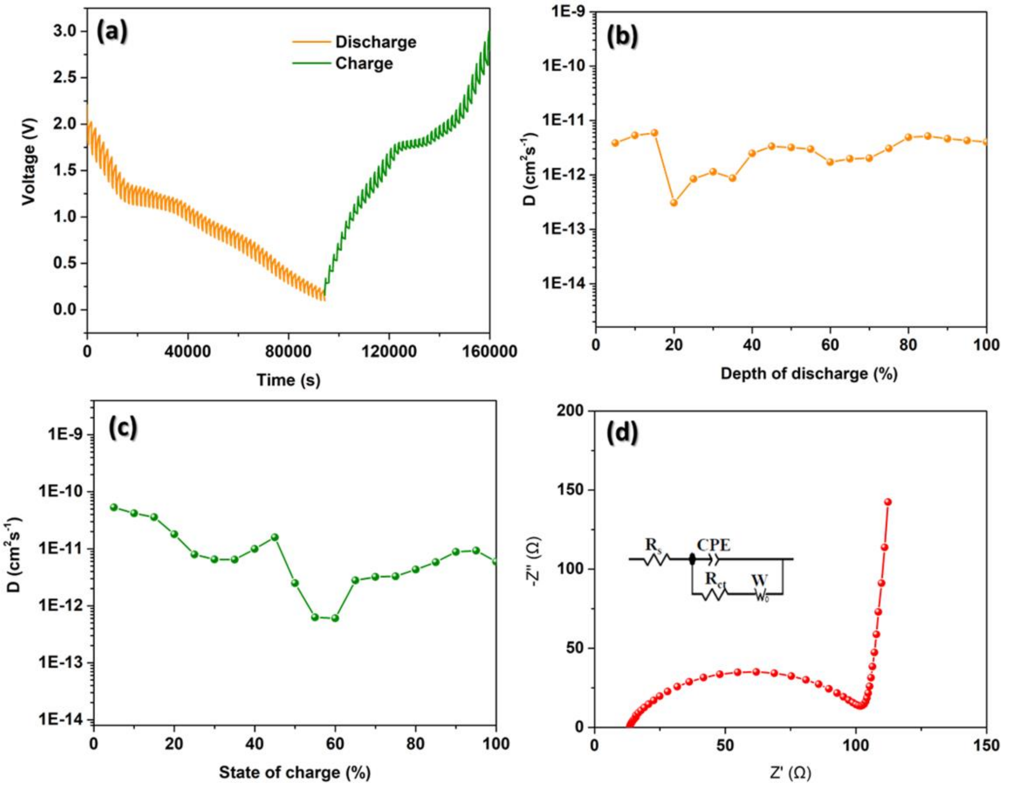

Figure S9 (a) GITT potential profiles of CoTe@Ti_3C_2. (b, c) Calculated Li^+ diffusion coefficients (D_{Li}) during the (b) discharge and (c) charge processes of CoTe@Ti_3C_2. (d) Nyquist plot from EIS of a fresh CoTe@Ti_3C_2 cell (inset: corresponding equivalent circuit model).

10. Radial distances of different atoms from the central Co atom in CoTe and Co crystal structure

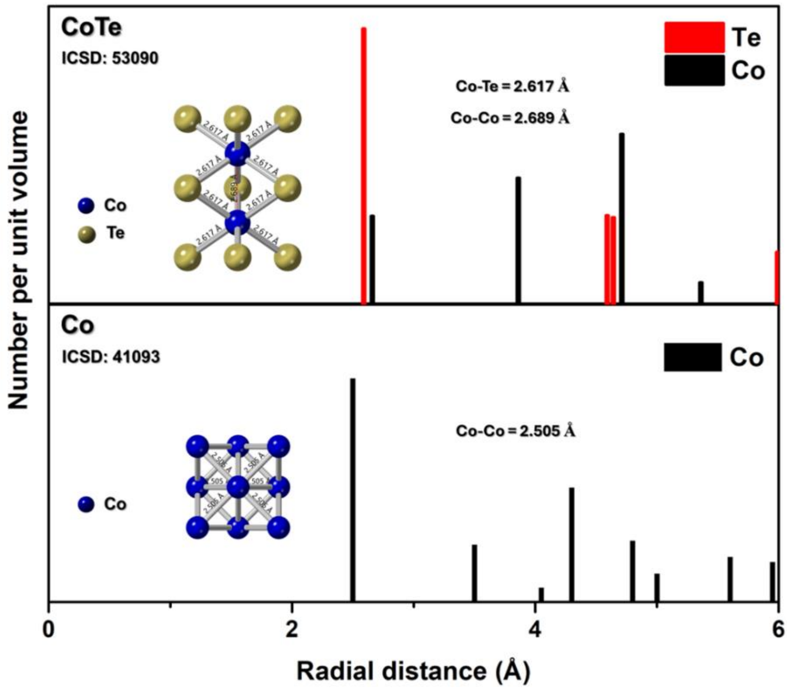

Figure S10 Radial distances of different atoms: (top) from the central Co atom in the CoTe crystal structure and (bottom) from the central Co atom in Co crystal structure.

11. *In-situ* Te K-edge XANES spectra of $CoTe@Ti_3C_2$ electrode

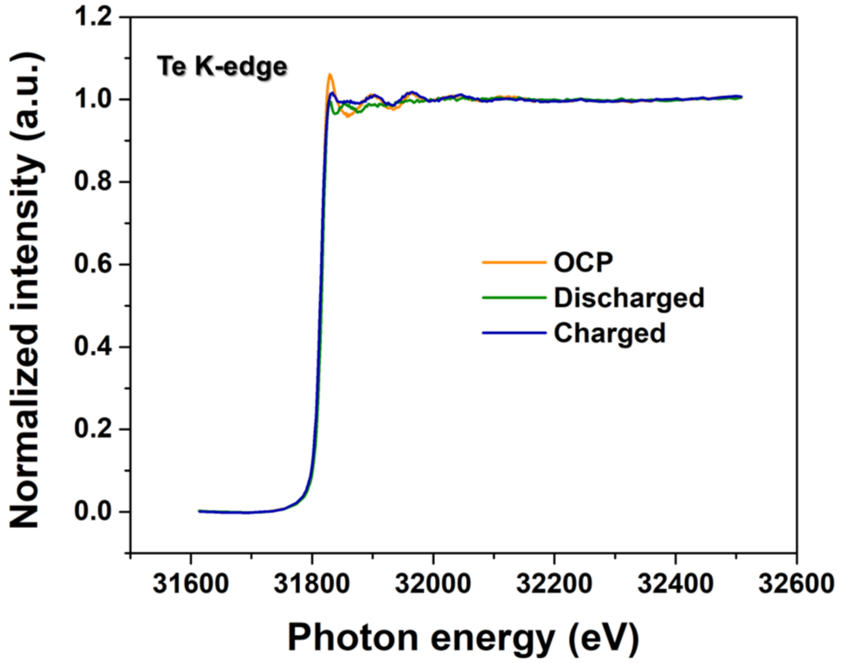

Figure S11 *In-situ* Te K-edge XANES spectra of $CoTe@Ti_3C_2$ electrode from the Li|LE|$CoTe@Ti_3C_2$ cell at OCP, discharged and charged states.

12. Full cell configuration and rate performance of the CoTe@Ti_3C_2//$LiFePO_4$

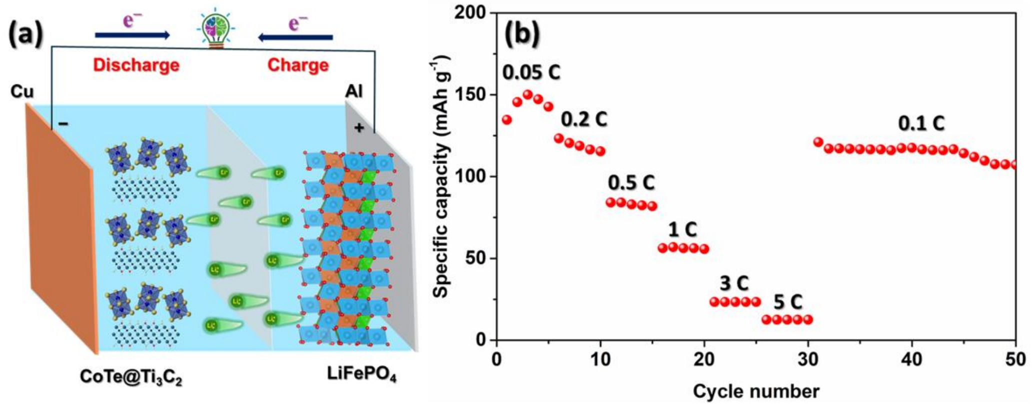

Figure S12 (a) Schematic illustration of the CoTe@Ti_3C_2//$LiFePO_4$ full cell LIB configuration. (b) Rate capability of the CoTe@Ti_3C_2-based full cell at various current densities.

13. *In-situ* XRD/XAS cell

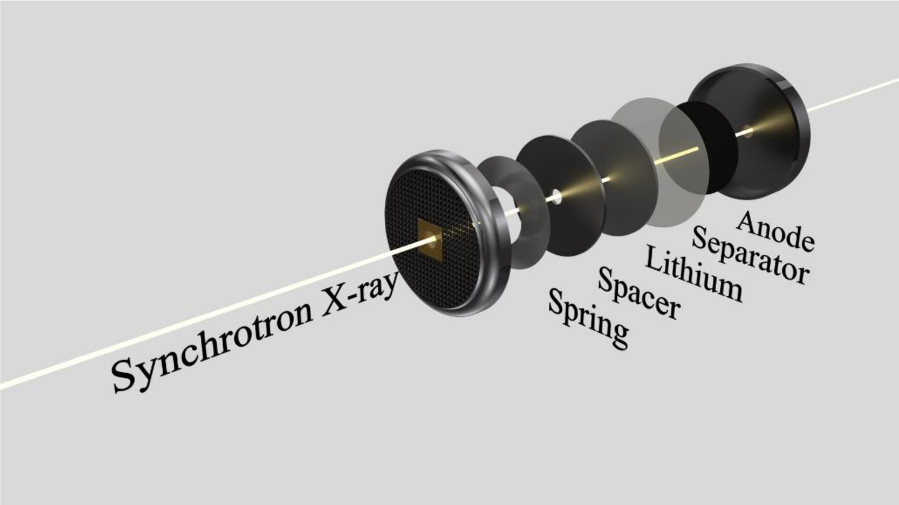

Figure S13 *In-situ* XRD/XAS cell.

14. Comparison of electrochemical performance of CoTe@Ti_3C_2 with other recently reported anode materials.

Active materials	Battery type	Current density	Specific capacity	Cycle stability	Refs.
Ti_3C_2/CoS_2	LIB	0.1 Ag^{-1}	405.8 mAh g^{-1} after 200 cycles	1000 cycles at 1.0 A g^{-1}	1
2H-$MoTe_2$	LIB	1.0 A g^{-1}	291 mAh g^{-1} after 260 cycles	260 cycles at 1.0 A g^{-1}	2
$CoTe_2$-C Nanocomposites	LIB<br>SIB	0.6 A g^{-1}<br>0.35 A g^{-1}	480 mAh g^{-1} after 200 cycles<br>250 mAh g^{-1} after 200 cycles	200 cycles at 0.6 A g^{-1}<br>200 cycles at 0.35 A g^{-1}	3
SiO_x/few layer graphene	LIB	0.05 Ag^{-1}<br>0.12 Ag^{-1}	350 mAh g^{-1} after 200 cycles<br>300 mAh g^{-1} after 200 cycles	200 cycles at 0.05 A g^{-1} and 0.12 A g^{-1}	4
$FeTe_2/CoTe_2$	LIB<br>SIB	0.1 Ag^{-1}<br>0.1 Ag^{-1}	350 mAh g^{-1} after 200 cycles<br>666 mAh g^{-1} after 200 cycles	100 cycles at 0.1 A g^{-1}<br>200 cycles at 0.1 A g^{-1}	5
$CoTe_2$	LIB	0.12 A g^{-1}	807 mAh g^{-1} after 200 cycles	200 cycles at 0.12 A g^{-1}<br>400 cycles at 0.6 A g^{-1}	6
Ti_3C_2 MXene/VN @C composite	LIB	0.1 A g^{-1}	645 mAh g^{-1} after 500 cycles	500 cycles at 0.1 A g^{-1}	7
CoTe@Ti_3C_2	LIB	0.1 A g^{-1}<br>1.0 A g^{-1}	698 mAh g^{-1} after 1000 cycles<br>213 mAh g^{-1} after 1300 cycles	1000 cycles at 0.1 A g^{-1}<br>1300 cycles at 1.0 A g^{-1}	This work

Table S1 Comparison of electrochemical performance of CoTe@Ti_3C_2 with other recently reported anode materials.

Reference

[1] S. Tian, D. Wang, Z. Liu, G. Liu, Q. Zeng, X. Sun, H. Yang, C. Han, K. Tao, S. Peng *ACS Appl. Mater. Interfaces* **2023**, *15*, 44996-45004.
[2] M. R. Panda, R. Gangwar, D. Muthuraj, S. Sau, D. Pandey, A. Banerjee, A. Chakrabarti, A. Sagdeo, M. Weyland, M. Majumder, Q. Bao, S. Mitra, *Small* **2020**, *16*, 2002669.
[3] V. Ganesan, K.-H. Nam, C.-M. Park, *ACS Appl. Energy Mater*. **2020**, *3*, 4877-4887.
[4] E. Barcaro, V. Marangon, M. Mutarelli, J. Hassoun, *J. Power Sources* **2024**, *595*, 234059.
[5] Zihao Li, Can Wan, Xijia Yang, Yue Yang, Xuesong Li, Yang Gao, Liying Wang, Wei Lü, *J. Power Sources* **2025**, *632*, 236361.
[6] H. Fan, C. Liu, G. Lan, P. Mao, R. Zheng, Z. Wang, Y. Liu*, H. Sun, *Electrochim. Acta* **2023**, *439*, 141614.
[7] Y. Wang, D. Zhang, B. Zhao, H. Chen, C. Chang, M. Liu, *ACS Appl. Nano Mater.* **2023**, *6*, 3572-3579.
